# Bone Metastasis in Renal Cell Carcinoma Patients: Risk and Prognostic Factors and Nomograms

**DOI:** 10.1155/2021/5575295

**Published:** 2021-05-12

**Authors:** Zhiyi Fan, Zhangheng Huang, Xiaohui Huang

**Affiliations:** ^1^Hangzhou Medical College, Hangzhou, Zhejiang Province, China; ^2^Department of Spine Surgery, Affiliated Hospital of Chengde Medical University, Chengde, Hebei Province, China

## Abstract

**Background:**

Bone metastasis (BM) is one of the common sites of renal cell carcinoma (RCC), and patients with BM have a poorer prognosis. We aimed to develop two nomograms to quantify the risk of BM and predict the prognosis of RCC patients with BM.

**Methods:**

We reviewed patients with diagnosed RCC with BM in the Surveillance, Epidemiology, and End Results (SEER) database from 2010 to 2015. Multivariate logistic regression analysis was used to determine independent factors to predict BM in RCC patients. Univariate and multivariate Cox proportional hazards regression analyses were used to determine independent prognostic factors for BM in RCC patients. Two nomograms were established and evaluated by calibration curve, receiver operating characteristic (ROC) curve, and decision curve analysis (DCA).

**Results:**

The study included 37,554 patients diagnosed with RCC in the SEER database, 537 of whom were BM patients. BM's risk factors included sex, tumor size, liver metastasis, lung metastasis, brain metastasis, N stage, T stage, histologic type, and grade in RCC patients. Currently, independent prognostic factors for RCC with BM included grade, histologic type, N stage, surgery, brain metastasis, and lung metastasis. The calibration curve, ROC curve, and DCA showed good performance for diagnostic and prognostic nomograms.

**Conclusions:**

Nomograms were established to predict the risk of BM in RCC and the prognosis of RCC with BM, separately. These nomograms strengthen each patient's prognosis-based decision making, which is critical in improving the prognosis of patients.

## 1. Introduction

Renal cell carcinoma (RCC) is one of the most common cancers worldwide, with approximately 403,262 new cases and 17,598 deaths in 2018 [1]. Approximately 15–30% of RCC patients have metastases at the initial diagnosis, and bone is a common site of metastasis [2, 3]. Bone metastasis (BM) from RCC is predominantly osteolytic and can lead to skeletal-related diseases, which can reduce the quality of life and prognosis of the patients [4, 5]. The median overall survival (OS) of RCC patients with BM has been reported to be only 12–28 months [6, 7]. In contrast, patients with metastatic RCC without BM had a more prolonged median OS to 31 months [8, 9]. Therefore, understanding the BM in RCC patients is an unmet need.

The TNM staging system is widely used to assess the prognosis of cancer patients, and clinicians use it to develop treatment plans [10]. Studies have shown that race, sex, age, and tumor size may also affect the prognosis of patients with RCC [11–13]. The TNM staging system relies on three pathological indicators and ignores other prognostic factors, thereby reducing the accuracy of prognostic prediction for RCC patients. Therefore, it is necessary to combine clinicopathology and other prognosis-related variables to construct a tool to accurately predict the prognosis and overcome the limitations of the traditional TNM staging system.

Nomogram is a tool that combines multiple biological and clinical variables to predict specific endpoints and has been widely used to predict the prognosis of cancer patients [14–16]. By combining these important variables, the nomograms can individually estimate the probability of events over time, such as the OS of cancer patients. In addition, nomograms can be used to estimate the survival rate of cancer patients with higher accuracy than the TNM staging system [17].

Risk factors and prognosis-related factors for BM in RCC have been reported in several previous studies [18–20]. However, no studies have focused on constructing predictive models for the risk and prognosis of BM in RCC, which means that the probability of outcome cannot be quantified. Therefore, based on the data from the Surveillance, Epidemiology, and End Results (SEER) database, we developed two nomograms for predicting the risk of BM with RCC and the OS of RCC patients with BM, separately.

## 2. Methods

### 2.1. Study Population Selection

The SEER database covers approximately 28% of cancer registries in the United States [21]. The data contained in this study were downloaded from the SEER ^*∗*^ Stat software version 8.3.6. Analysis of anonymous data from the SEER database is exempt from medical ethics review and does not require informed consent. The SEER database provides clinical information on cancer patients that greatly facilitate clinical research. Patients diagnosed before 2010 were excluded because the SEER database did not record information on distant metastases until 2010. In addition, to ensure adequate follow-up time, patients diagnosed after 2015 are not included. Therefore, only patients diagnosed with RCC between 2010 and 2015 were considered in this study.

Inclusion criteria were as follows: (1) RCC as the first primary tumor, (2) patients with a histologic diagnosis of RCC, and (3) patients with complete clinicopathological features, demographic information, and follow-up information. In addition, patients who were certified by autopsy or death were excluded from this study. Finally, a total of 37,554 patients with RCC were enrolled to study the risk factors for BM in patients with RCC and to establish a diagnostic nomogram. Subsequently, for RCC patients with BM with survival time ≥ one month, specific treatment information, including surgery, radiotherapy, and chemotherapy, were used to form a new cohort to explore the prognostic factors for RCC patients with BM and develop a prognostic nomogram. Ultimately, 537 patients were used to study prognostic factors in patients with BM from RCC. Patients in each cohort were randomized into training and validation cohorts in a 7 : 3 ratio. In this study, patients in the training cohort were used to construct the predicted nomogram, while patients in the validation cohort were used to validate the constructed nomogram.

### 2.2. Data Collection

Based on patient-specific information from the SEER database, we selected 14 variables to identify risk factors for BM in RCC, including age, sex, race, tumor size, histologic type, grade, laterality, T stage, N stage, distant metastatic site (lung, brain, liver), insurance status, and marital status. In addition to the aforementioned variables, information on surgery, radiotherapy, and chemotherapy are included to study the factors that influence the prognosis of RCC patients with BM. The optimal cutoff values for tumor size in terms of OS were determined by X-tile software, and patients were divided into three groups (<4, 4–7, and >7 cm). The histologic type was defined by the following ICD-O-3 codes: clear cell (8310/3, 8313/3), papillary (8260/3), chromophobe (8317/3, 8270/3), and collecting duct (8319/3). Regarding marital status, we excluded misleading data on unmarried or domestic partners and then included “unmarried,” “separated,” “single,” and “widowed” all in the unmarried group. Insurance status is divided into insured and uninsured, with both “insured” and “insured/unspecific” included in the insured group. All cases in this study were staged using version 7 of the American Joint Committee on Cancer TNM staging system. In the survival analysis, the primary endpoint of our study was OS, which was defined as the date from diagnosis to death (for any reason) or the date of the last follow-up.

### 2.3. Statistical Analysis

This study used SPSS 25.0 and R software (version 3.6.1) for statistical analysis. The chi-square test was used for categorical data. Variables with *P* values <0.05 in univariate analysis were incorporated into a multivariate logistic regression analysis to identify independent risk factors for BM in RCC patients. At the same time, univariate Cox proportional hazards regression analysis was used to determine OS-related variables. Significant variables in the univariate Cox proportional hazards regression analysis were then included in the multivariate Cox proportional hazards regression analysis to identify independent prognostic factors in RCC patients with BM.

Nomograms were developed separately based on independent BM-related predictors and prognostic factors using the “rms” package in R software. In the nomograms, values for the individual patient were located along the variable axes, and a line was drawn upward to the points axis to determine the number of points assigned for each variable. There was a total points line at the bottom of the nomogram, and each variable score was summed to give the total points. Receiver operating characteristic (ROC) curves for two nomograms were generated, and the corresponding area under the curve (AUC) was used to evaluate the discrimination of nomograms. The clinical application value of the nomogram model was evaluated by calibration curve and decision curve analysis (DCA). Finally, all patients were divided into high-risk and low-risk groups according to the median of risk score, and survival curves were used to verify the prognostic value of the nomogram [22].

## 3. Results

### 3.1. The Characteristics of the Study Population

**Figure 1:**
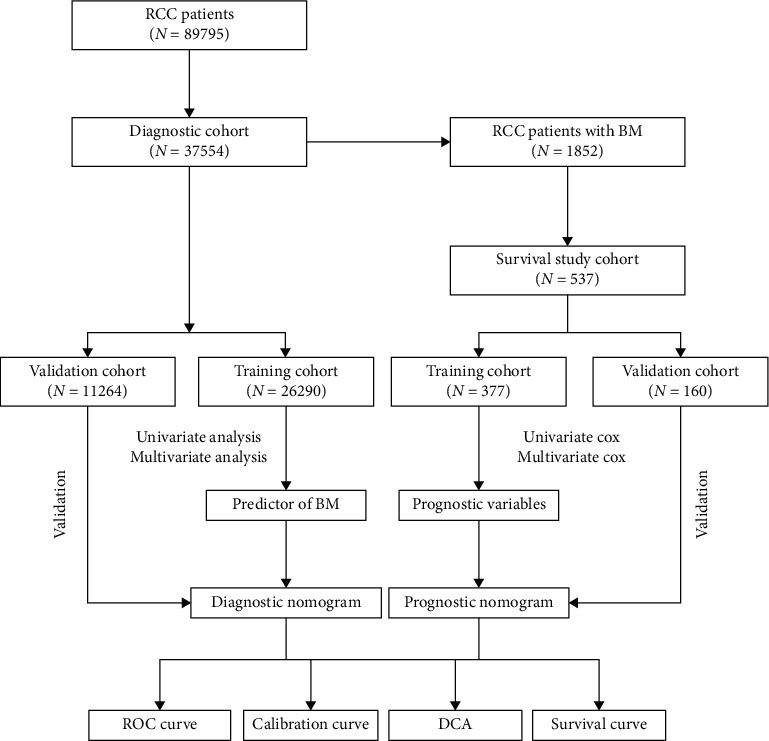
The workflow describing the schematic overview of the project.

The workflow of our study is illustrated in Figure 1. A total of 37,554 RCC patients from the SEER database were included. Furthermore, 26,290 and 11,264 patients were included in the training and validation cohorts, respectively. Clinicopathological information of 26,290 RCC patients is given in Table 1.

### 3.2. Risk Factors of BM in RCC Patients

To identify BM-related variables in RCC patients, 14 factors were analyzed. The results showed that ten factors were related to the BM in RCC patients, including race, sex, grade, histologic type, T stage, N stage, brain metastasis, liver metastasis, lung metastasis, and tumor size (Table 1). Subsequently, the above variables were included in the multivariate logistic regression analysis, which showed that tumor size, liver metastasis, lung metastasis, brain metastasis, N stage, T stage, histologic type, and grade were independent predictors of RCC with BM (Table 2).

### 3.3. Development and Validation of a Nomogram for BM in Newly Diagnosed RCC Patients

**Figure 2:**
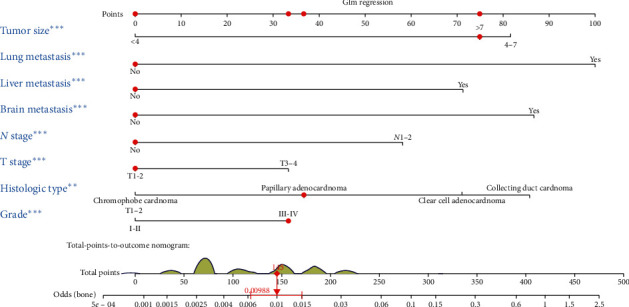
Nomogram to estimate the risk of BM in patients with RCC.

**Figure 3:**
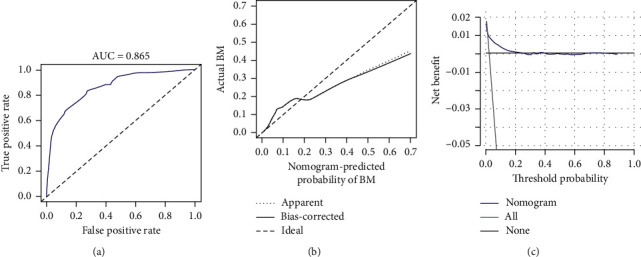
ROC curves (a), calibration curves (b), and DCA (c) of the training cohort.

**Figure 4:**
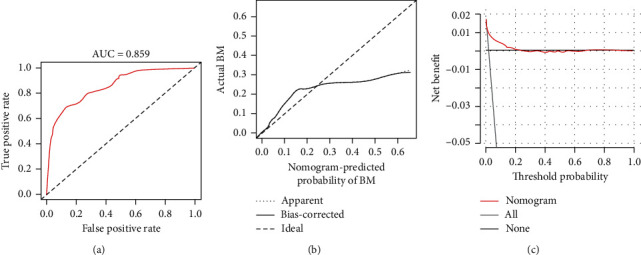
ROC curves (a), calibration curves (b), and DCA (c) of the validation cohort.

**Figure 5:**
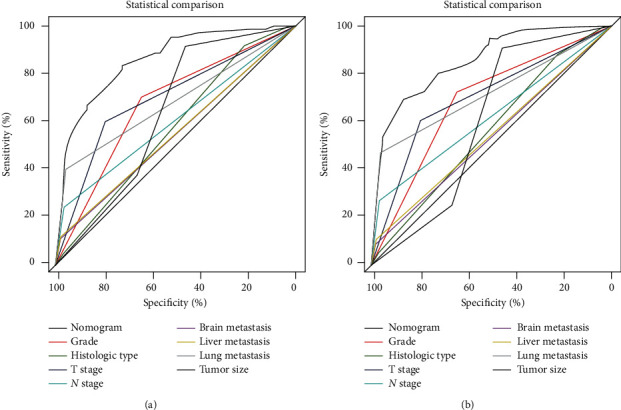
Comparison of AUC between diagnostic nomogram and each independent predictor in the training cohort (a) and the validation cohort (b).

Based on eight independent BM-related variables, a nomogram was constructed to assess the risk of BM in RCC patients (Figure 2). The AUCs of the nomogram were 0.865 and 0.859 in the training and validation cohorts, respectively, showing good discrimination (Figure 3(a) and Figure 4(a)). The calibration curve showed that the observations are highly consistent with the predicted results (Figure 3(b) and Figure 4(b)). Moreover, DCA indicated that the diagnostic nomogram performs well in clinical practice (Figure 3(c) and Figure 4(c)). Importantly, ROC curves were generated for each independent predictor variable. As shown in Figure 5, the AUC of the nomogram is higher than the AUCs of all independent variables in both training and validation cohorts, indicating a significant advantage in the accuracy of predictions using the nomogram compared to predictions using individual independent predictors.

### 3.4. Prognostic Factors for RCC Patients with BM

**Figure 6:**
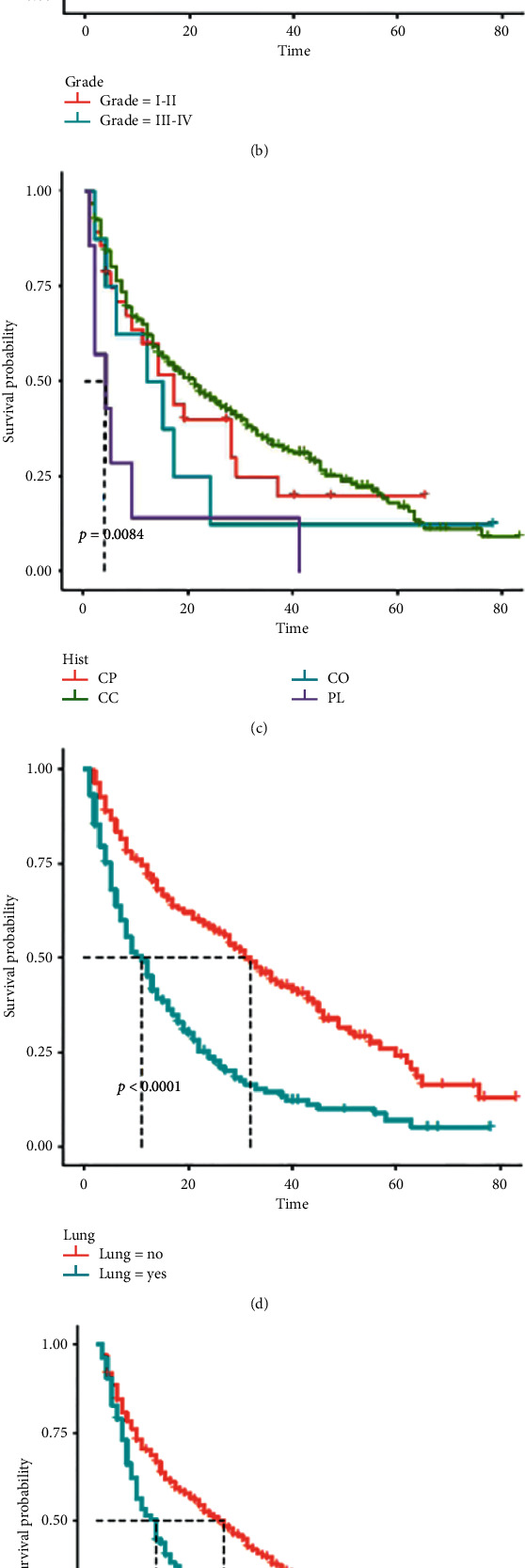
Survival curves for each independent prognostic factor in the training cohort. CP, chromophobe; CC, clear cell; CD, collecting duct; PL, papillary.

According to the selection process, a total of 537 patients with BM were included in our research. Meanwhile, 377 patients were incorporated into the training cohort, and the remaining 160 patients were incorporated into the validation cohort. Univariate and multivariate Cox proportional hazards regression analyses were performed to screen for prognostic factors. Univariate Cox proportional hazards regression analysis showed that grade, T stage, histologic type, N stage, surgery, chemotherapy, brain metastasis, liver metastasis, and lung metastasis are OS-related factors (Table 3). After controlling for confounding variables using multivariate Cox proportional hazards regression analysis, grade, histologic type, N stage, surgery, brain metastasis, and lung metastasis were identified as independent prognostic factors in RCC patients with BM (Table 3). As shown in Figure 6, the survival curve analysis further demonstrated the impact of screened independent prognostic factors on the OS of RCC patients with BM.

### 3.5. Prognostic Nomogram for RCC Patients with BM

**Figure 7:**
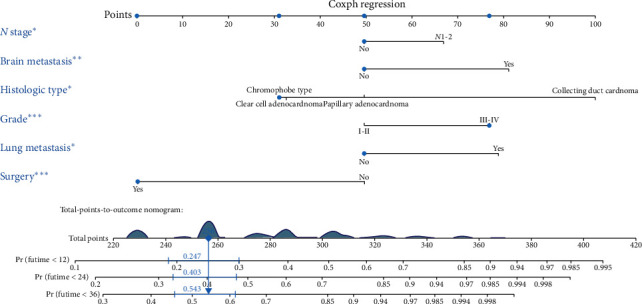
Nomogram to predict the OS of RCC patients with BM.

**Figure 8:**
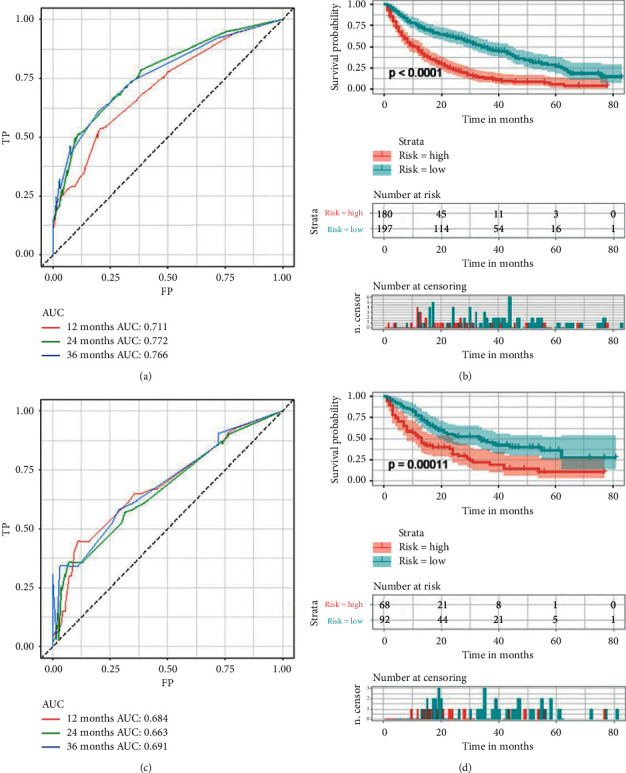
(a) Receiver operating characteristic curves of 1, 2, and 3 years in the training cohort. (b) The Kaplan–Meier survival curve of the training cohort. (c) Receiver operating characteristic curves of 1, 2, and 3 years in the validation cohort. (d) The Kaplan–Meier survival curve of the validation cohort.

**Figure 9:**
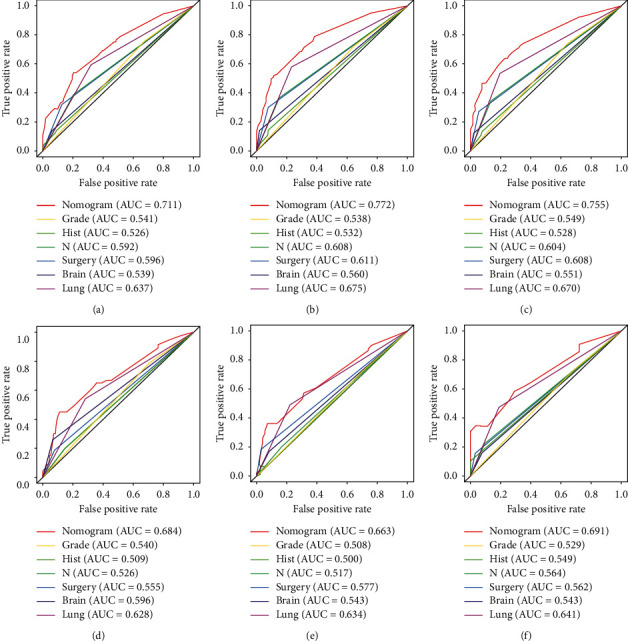
The receiver operating characteristic curves of nomogram and all independent predictors at 1 (a), 2 (b), and 3 years (c) in the training cohort and at 1 (d), 2 (e), and 3 years (f) in the validation cohort.

**Figure 10:**
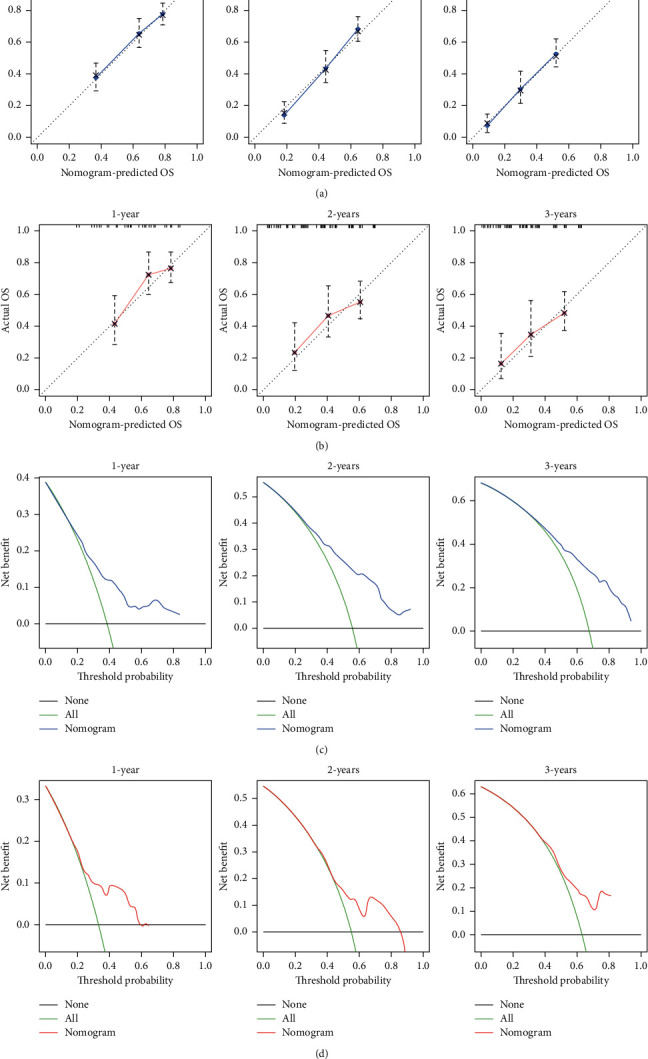
(a) The calibration curves of the prognostic nomogram in the training cohort. (b) The calibration curves of the prognostic nomogram in the validation cohort. (c) The decision curve analysis of the prognostic nomogram in the training cohort. (d) The decision curve analysis of the prognostic nomogram in the validation cohort.

A prognostic nomogram of RCC patients with BM based on six independent prognostic factors was established (Figure 7). The ROC curve showed that the AUCs at 1, 2, and 3 years were 0.711, 0.772, and 0.766 in the training cohort and 0.684, 0.663, and 0.691 in the validation cohort (Figure 8(a) and 8(c)). The optimal cutoff point for the total score was determined by X-tile software and was 285. Therefore, we specified less than 285 as the low-risk group and greater than 285 as the high-risk group. By depicting the Kaplan–Meier survival curve, we can find that patients in the high-risk group showed a worse prognosis than patients in the low-risk group (Figure 8(b) and 8(d)). In addition, we further compared the discrimination between the nomogram and the independent prognostic factors, and the results showed that the AUC of the nomogram was higher than the AUCs of all independent factors at 1, 2, and 3 years, both in the training cohort and in the validation cohort (Figure 9). Calibration curves of predicting 1, 2, and 3-year OS probabilities also show good agreement between the OS predicted by the prognostic nomogram and the actual results (Figure 10(a) and 10(b)). The DCA was used to evaluate the clinical utility of a nomogram. As shown in Figure 10, the prognostic nomogram shows a significant positive net benefit over a wide range of mortality risks, suggesting its high clinical utility in predicting OS in RCC patients with BM.

## 4. Discussion

RCC accounts for 3% of all malignancies and 80%–85% of primary renal cancer [23]. Bone is the second most common site of metastasis in RCC patients, following the lung [24, 25]. In the present study, we constructed diagnostic and prognostic nomograms to predict the risk of BM in RCC patients and the OS of RCC patients with BM by analyzing massive data, respectively. We believe that two nomograms representing OS and distant metastasis are complementary and can increase their clinical value in patients with RCC. The total score can be calculated by obtaining data for each RCC patient's corresponding variable on the nomogram. The risk of BM can then be easily identified on the diagnostic nomogram, identifying patients in the high-risk group and guiding clinical practice in early intervention. Similarly, the prognosis of RCC patients with BM can be determined from the prognostic nomogram. In the validation of the two nomograms, the two nomograms showed excellent performance in BM risk assessment and OS prediction in RCC patients, respectively, which will enable more accurate personalized clinical decision making and monitoring.

Despite the poor prognosis of RCC patients with BM, early detection of BM may be critical for patients with RCC to receive appropriate treatment. Therefore, exploring the risk factors for BM in RCC patients is important for clinical decision making. At the molecular level, cadherin-11, transforming growth factor-*β*, insulin-like growth factor, and the fibroblast growth factor have been associated with BM in RCC patients [26, 27]. Nevertheless, these biomarkers are difficult and impractical to apply immediately to clinical decision making. In our daily clinical work, it is difficult for us to examine every patient at the molecular level because it requires a lot of human and material resources. At the same time, the high cost of testing at the molecular level is difficult for patients to afford. Of course, if molecular level indicators could be included in the nomogram, this would undoubtedly increase the predictive accuracy of the nomogram, which could lead to better survival for patients. In addition, regarding some practical clinical features, sex, T stage, N stage, grade, liver metastasis, lung metastasis, brain metastasis, and histologic type have been reported as relevant risk factors for BM in RCC [20]. However, to date, no predictive model has been developed, which means that it is impossible to identify an individual's risk of BM by combining all independent predictors associated with BM. The present study showed that tumor size, liver metastasis, lung metastasis, brain metastasis, N stage, T stage, histologic type, and the grade were significant predictors of BM in RCC. The association between these factors and BM in RCC patients has been reported in previous studies. Although metastasis to multiple organs is a risk factor for BM in patients with RCC, unfortunately, we were unable to obtain the sequence of organ metastasis due to the shortcomings of the SEER database itself. Previous studies have confirmed the relationship between tumor grade, TNM staging, and BM in RCC patients [20]. TNM staging is widely used in the assessment of prognosis in cancer patients. Notably, a more significant contribution of TNM staging was shown in both the diagnostic nomogram and the prognostic nomogram. With increasing tumor size, an increasing number of lymph node metastases, and distant organ metastases, the risk of BM in RCC and the risk of death in RCC patients with BM are significantly increased.

In addition, our study found a poor prognosis of patients with lymph node metastasis, brain metastasis, lung metastasis, without surgery, poor tumor differentiation, and histologic type of the collecting duct. A prognostic nomogram was established based on six independent prognostic factors. The results suggested that a nomogram can be an effective tool for identifying high-risk patients. The impact of histologic type on metastatic potential and prognosis of metastatic patients is often overlooked when discussing treatment options. In this study, collecting duct RCC had a higher incidence of BM and a worse prognosis compared to other renal cancer subtypes. Collecting duct RCC is reported to be a rare entity that occurs in <2% of patients with kidney cancer, often resulting in a poor prognosis [28]. In addition, the above correlation has been confirmed in previous studies [29, 30]. The relationship between lung metastasis, brain metastasis, surgery, and prognosis in patients with RCC has also been widely reported in previous studies. Lin et al. reported a better prognosis in patients with only BM than in patients with concomitant pulmonary metastases and a significantly better prognosis for patients with single BM than in patients with multiple bones and/or visceral metastasis [31]. Similarly, Toyoda et al. reported a shorter median survival in patients with extra-BM compared to those without (8 vs. 33 months, *P* = 0.0084) [32]. Surprisingly, contrary to previous reports, the presence of liver metastasis was not an independent prognostic factor in our study [33, 34]. However, this is consistent with what has been reported by Santoni et al. [19]. Previous studies have reported age as a factor associated with patient prognosis regarding RCC, but other studies show no difference in prognosis between younger and older patients with RCC [35, 36]. Some of these studies included only a restricted age group of patients or limited sample size or follow-up time. Thus, until now, the role of age as a prognostic factor in patients with RCC has been controversial. The study included as many factors as possible that may be associated with the prognosis of patients with RCC and identified the relevant prognostic factors by rigorous statistical methods, so the results are trustworthy. However, due to the retrospective nature of the study, selection bias is inevitable. For the treatment of RCC patients with BM, recent consensus suggests using a multimodal treatment strategy that includes extensive resection of the lesion, radiotherapy, systemic therapy, and other local treatment options [37]. Of RCC patients with BM, surgical treatment aims to improve the prognosis, local tumor control, pain relief, and preservation or reconstruction of function. Based on the results, we found that surgery was not only an independent prognostic factor but that patients who had surgery showed a better prognosis. As reported in several studies, surgical removal of isolated or minimally metastatic lesions can improve the prognosis of patients with BM, thus providing a multidisciplinary team to support the treatment plan for these patients [38–40]. Although renal cancer is usually not sensitive to radiotherapy and chemotherapy, palliative radiotherapy can significantly relieve local symptoms and improve quality of life [41, 42]. Tyrosine kinase inhibitors (TKIs) and antivascular endothelial growth factor antibodies are now widely used as first- and second-line therapy for advanced RCC. Direct evidence on the effects of targeted drugs on BM is currently limited to a few studies that have shown that TKIs can prolong the mean time to progression of existing bone lesions and reduce the formation of new bone lesions [2, 43]. Unfortunately, the SEER database does not contain specific analyses of targeted therapies, chemotherapy, and radiotherapy, and we are unable to analyze their influence on prognosis in further detail. In addition, further research on important prognostic factors for OS with BM in RCC is necessary.

However, some limitations of our study should be noted. First, information collected in the SEER database is about the disease at the first diagnosis and does not record BM that occurred later. Second, the prognostic impact of the amount of BM should not be overlooked, but there is no record of this in the SEER database. Third, we did not have access to some biomarkers from the SEER database, such as transforming growth factor-*β*, insulin-like growth factor, and fibroblast growth factor. Fourth, this was a retrospective study in which selection bias was inevitable, and detailed treatment was not available in the SEER database. Immunotherapy was recommended for patients with RCC because of its OS benefit, but, unfortunately, the SEER database does not contain this information. As such, the validity of this data is not any more known. In addition, since the construction and validation cohorts are from the same database, it is still necessary to validate the accuracy of the nomograms in other databases.

## 5. Conclusions

Two nomograms we created could be used as a supportive graphic tool in RCC patients to help clinicians distinguish, assess, and evaluate the risk and prognosis of RCC with BM. At the same time, when faced with individualized condition consultation, these nomograms are valuable methods to provide prognostic information to clinical patients and strengthen each patient's prognosis-based decision making, which is of great significance in improving the prognosis of patients.

## Figures and Tables

**Table 1:** Demographic and clinical characteristics of RCC patients.

	Without BM	With BM	*χ* ^2^	*P*
Age			1.463	0.226
<65	14388 (55.7%)	264 (58.5%)		
≥65	11451 (44.3%)	187 (41.5%)		

Sex			6.982	0.008
Female	9111 (35.3%)	132 (29.3%)		
Male	16728 (64.7%)	319 (70.7%)		

Race			6.289	0.043
Black	2648 (10.2%)	30 (6.7%)		
Others	1585 (6.1%)	28 (6.2%)		
White	21606 (83.6%)	393 (87.1%)		

Grade			236.193	≤0.001
I-II	16818 (65.1%)	136 (30.2%)		
III-IV	9021 (34.9%)	315 (69.8%)		

T Stage			437.434	≤0.001
T1-2	20760 (80.3%)	182 (40.4%)		
T3-4	5079 (19.7%)	269 (59.6%)		

Laterality			1.692	0.193
Left	12723 (49.2%)	236 (52.3%)		
Right	13116 (50.8%)	215 (47.7%)		

Histologic type			79.734	≤0.001
CP	1416 (5.5%)	6 (1.3%)		
CC	20244 (78.3%)	407 (90.2%)		
CD	54 (0.2%)	7 (1.6%)		
PL	4125 (16.0%)	31 (6.9%)		

Tumor size, cm			392.797	≤0.001
<4	12087 (46.8%)	39 (8.6%)		
4–7	8559 (33.1%)	167 (37.0%)		
>7	5193 (20.1%)	245 (54.3.0%)		

*N* stage			733.940	≤0.001
N0	25218 (97.6%)	345 (76.5%)		
N1	621 (2.4%)	106 (23.5%)		

Brain metastasis			679.401	≤0.001
No	25741 (99.6%)	408 (90.5%)		
Yes	98 (0.4%)	43 (9.5%)		

Liver metastasis			629.229	≤0.001
No	25707 (99.5%)	404 (89.6%)		
Yes	132 (0.5%)	47 (10.4%)		

Lung metastasis			1835.076	≤0.001
No	25139 (97.3%)	274 (60.8%)		
Yes	700 (2.7%)	177 (39.2%)		

Insurance status			0.064	0.800
No	750 (2.9%)	14 (3.1%)		
Yes	25089 (97.1%)	437 (96.9%)		

Marital status			3.481	0.062
No	8109 (31.4%)	123 (27.3%)		
Yes	17730 (68.6%)	328 (72.7%)		

BM, bone metastasis; RCC, renal cell carcinoma; CP, chromophobe; CC, clear cell; CD, collecting duct; PL, papillary.

**Table 2:** Multivariate logistic regression analysis of BM in RCC patients.

Variables	OR (95% CI)	*P* value
Grade		
G1-2	Reference	
G3-4	1.749 (1.388–2.204)	≤0.001

T stage		
T1-2	Reference	
T3-4	1.748 (1.379–2.216)	≤0.001

Histologic type		
CP	Reference	
CC	3.300 (1.459–7.466)	0.004
CD	4.216 (1.245–14.277)	0.021
PL	1.850 (0.762–4.490)	0.174

Tumor size, cm		
<4	Reference	
4–7	3.937 (2.745–5.648)	≤0.001
>7	3.510 (2.374–5.189)	≤0.001

N stage		
N0	Reference	
N1	2.654 (2.005–3.513)	≤0.001

Brain metastasis		
No	Reference	
Yes	4.283 (2.780–6.598)	≤0.001

Liver metastasis		
No	Reference	
Yes	3.309 (2.211–4.952)	≤0.001

Lung metastasis		
No	Reference	
Yes	5.351 (4.123–6.946)	≤0.001

BM, bone metastasis; RCC, renal cell carcinoma; CP, chromophobe; CC, clear cell; CD, collecting duct; PL, papillary.

**Table 3:** Univariate and multivariate Cox analyses in RCC patients with BM.

	Univariate Cox analysis				Multivariate Cox analysis			
	HR	95% CI		*P*	HR	95% CI		*P*
Age								
<65								
≥65	1.026	0.798	1.319	0.844				

Race								
Black								
Others	1.660	0.913	3.020	0.097				
White	1.293	0.827	2.023	0.260				

Sex								
Female								
Male	1.109	0.847	1.452	0.451				

Grade								
I-II								
III-IV	1.411	1.074	1.853	0.013	1.669	1.235	2.257	≤0.001

T stage								
T1-2								
T3-4	1.388	1.080	1.785	0.010				

Laterality								
Left								
Right	1.099	0.864	1.397	0.443				

Histologic type								
CP								
CC	0.848	0.537	1.339	0.479	0.706	0.440	1.132	0.148
CD	1.157	0.487	2.746	0.742	0.723	0.300	1.743	0.471
PL	2.767	1.167	6.557	0.021	2.492	1.036	5.994	0.041
Tumor size, cm								
<4								
4–7	1.125	0.683	1.851	0.644				
>7	1.323	0.821	2.132	0.250				

N stage								
N0								
N1	1.791	1.378	2.328	≤0.001	1.388	1.049	1.838	0.022

Surgery								
No								
Yes	0.416	0.313	0.552	≤0.001	0.394	0.284	0.546	≤0.001

Radiotherapy								
No								
Yes	1.103	0.862	1.411	0.438				

Chemotherapy								
No								
Yes	1.463	1.128	1.898	0.004				

Brain metastasis								
No								
Yes	2.315	1.575	3.403	≤0.001	1.801	1.201	2.700	0.004

Liver metastasis								
No								
Yes	1.960	1.349	2.847	≤0.001				

Lung metastasis								
No								
Yes	2.261	1.771	2.887	≤0.001	1.745	1.342	2.269	≤0.001

Insurance status								
No								
Yes	1.227	0.628	2.396	0.549				

Marital status								
No								
Yes	0.994	0.768	1.286	0.961				

BM, bone metastasis; RCC, renal cell carcinoma; CP, chromophobe; CC, clear cell; CD, collecting duct; PL, papillary.

## Data Availability

The dataset from the SEER database that was generated and/or analyzed during the current study is available in the SEER dataset repository (https://seer.cancer.gov/).

## Abbreviations

AUC: Area under the curve
BM: Bone metastasis
DCA: Decision curve analysis
OS: Overall survival
ROC: Receiver operating characteristic
RCC: Renal cell carcinoma
SEER: Surveillance, Epidemiology, and End Results
TKIs: Tyrosine kinase inhibitors.
